# Spectral characteristics of postural sway in diabetic neuropathy patients participating in balance training

**DOI:** 10.1186/2251-6581-12-29

**Published:** 2013-06-19

**Authors:** Hoda Salsabili, Farid Bahrpeyma, Ali Esteki, Mansureh Karimzadeh, Hamed Ghomashchi

**Affiliations:** 1 Department of Physical Therapy, Tarbiat Modares University (School of medical Sciences), Tehran, Iran; 2 Department of Biomedical Engineering, Shahid Beheshti University, Tehran, Iran; 3 Department of Pre School education, University of Social Welfare and Rehabilitation, Tehran, Iran; 4 Faculty of Industrial and Mechanical Engineering, Islamic Azad University, Qazvin Branch, Qazvin, Iran

**Keywords:** Diabetic neuropathy, Balance training, Spectral characteristic, Total frequency power, Visual feedback, Postural sway

## Abstract

**Background:**

The aim of the present study is to have a detailed frequency analysis about the effect of balance training with respect to reactive movement strategies and sensory strategies in type 2 diabetic neuropathy (DN) patients. Also understand changes in the role of each postural subsystem for controlling quiet standing after balance training.

**Methods:**

A group of 19 patients were included in the quasi experimental, time- series study. Total frequency power, 99% power frequency, centroidal frequency and frequency spectrum in the intervals between 0.01-0.1, 0.1-0.5, 0.5-1 Hz and 1-3 Hz are reported. The training protocol consisted two patterns of limits of stability trainings, three approaches in weight shifting trainings and one stable standing practice on the biodex stability system. Results: Repeated measure ANOVA analysis and the LSD test indicated significant differences for the eyes open ML- frequency power and ML-FFT sway power within low-medium (0.1-0.5 HZ) frequencies.

**Conclusions:**

Decrease in postural sway at low-medium frequencies showed lower reliance on vestibular system. Also, better controlling hip muscles after balance training relieve DN patients’ requirement to more exploratory sway as a compensatory strategy and showed better balance performance after balance training in DN patients.

**Trial registration:**

UMIN-CTR Search Clinical Trials: UMIN000004485.

## Background

Complex interactions between postural subsystems which include proprioception, vestibular and visual systems maintain upright posture [1] and manifested in postural sway parameters [2]. Relationship between type 2 diabetic neuropathy (DN) and postural sway show balance problems are attributed to sensory ataxia [3] which is the lack of accurate proprioceptive feedback [4]. Thus, loss or reduction of peripheral sensory information from the feet [5,6] and the inability of the central nervous system (CNS) to appropriately integrate available postural control information [3,5,7] causes postural instability in neuropathic patients with type 2 diabetes. Additionally, switch from an ankle-based to a hip-based balance strategy [7-11], increase in the use of vestibular information [12] and pick up more useful information as exploratory sway at the hip level [5] changes the mechanism of postural control in diabetic neuropathy patients.

Balance training was reported as a useful method to improve DN patients’ inabilities in controlling posture. In this way, studies showed that physiotherapeutic group training such as gait and balance exercises with function orientated strengthening exercises [13], structured balance exercises [14] and distal strength and balance training [15] has the ability to describe balance training effectiveness in DN patients. Also, studies about the effects of dynamic balance training on quiet standing control in DN patients showed improvement in medial-lateral median and mean frequency of postural sway after balance training which showed effective balance training may treat context-specific instabilities of DN patients’ postural control [16]. Context-specific balance training refers to training which recruit reactive movement strategies and sensory strategies by exposing the patients to external perturbations with small movements of sway, like an inverted pendulum as an ankle strategy, and hip strategy by quick and narrow movements of the center of mass [16,17].

Despite the numerous researches on DN patients’ neuropathy balance training and reported study about alteration in postural sway of DN patients which indicated an increase in sway power density within medium-high frequencies (0.5-1.00 HZ) show lower postural control in diabetic neuropathy patients [18]. We found lack of frequency analyses in the field of alterations in DN patients’ postural control after balance training. For instance, in this study frequency domain analysis expanded in more details to represent the data as a function of frequencies [19] about the postural sway of DN patients after balance training. Benefits of the Fourier Spectral Analysis of postural sway have been explored in several independent studies. These studies have shown that typical ranges of postural frequency (i.e. frequency bands) express the different levels of activity of postural subsystems which may affect postural sway. Study of postural frequency can provide insights into the individual’s use of these postural subsystems. For example, Some Different independent studies in Fourier transform analyses revealed that low frequencies (0.01-0.1 Hz) show visual control, the low-medium frequency band (0.1-0.5 Hz) is sensitive to vestibular stress and disturbance, the medium-high frequencies (0.5-1 Hz) link to somatosensory activity and postural reflexes mediated by lower extremities and over 1 Hz frequency band are induced by dysfunction in the central nervous system [20-24]. Indeed, frequency domain analysis helps us to discriminate between patterns with similar time domain but different frequency domain [19] and might be a valuable tool in clinical diagnosis [18].

Therefore, we hypothesize that balance training with respect to reactive movement strategies and sensory strategies in DN patients’ can improve postural control and we can specifically show the changes by frequency analysis. Also we can understand differences in the role of each postural subsystem (visual, vestibular and somatosensory) by analyzing the spectral characteristics of center of pressure (COP) fluctuations after balance training.

## Methods

### Participants

A group of 19 patients (12 women and 7 men) with type 2 diabetic neuropathy were included in the non randomized, quasi experimental and time- series study (Table 1). Patients’ selection was based on several criteria: 1) having controlled type 2 diabetes for more than 5 years, 2) age between 40-70 years, 3) Fasting Blood Sugar (FBS) test results more than 110 mg/dl and less than 200 mg/dl, 4) Valk neuropathy score greater than 2, 5) A1c higher than 8.5, and 6) Snellen visual chart score of more than 16/20 [2]. Patients’ neuropathy was evaluated by recording nerve conduction velocity (NCV), amplitude and latency tests of sensory (sural) and motor (proneal and tibial) nerves. The motor or sensory nerve conduction velocity of 39 m/s was adopted as the lower limit (cut off value: below average – 2 SD of the normal range) [2,25]. Participants were excluded if they showed orthopedic or neurological disorders related to balance performance, hypotension and autonomic neuropathy [2], retinopathy, scars on the soles of their feet or any prior experience of similar balance trainings using the biodex stability system (BSS), force platform and tilt board.

**Table 1:** Patients baseline descriptive and demographic characteristics

**Demographic information**	**Patients**
Sex Ratio	12 women and 7 men
Age (years)	56 ± 8.96
Height (cm)	164.52 ± 5.09
Weight (kg)	76.52 ± 12.87
BMI (Kg/m^2^)	28.33 ± 4.65
Diabetic History (years)	11.84 ± 6.93

Values are Mean ± SD.

Patients were completely informed about the purposes of the study and each patient signed a consent form prior to participation. The ethical committee of Tarbiat Modares University which is in accordance with the revised declaration of Helsinki in 2000 approved the study protocol and informed consent forms.

### Experimental procedure

#### Assessments and measurements

**Figure 1:**
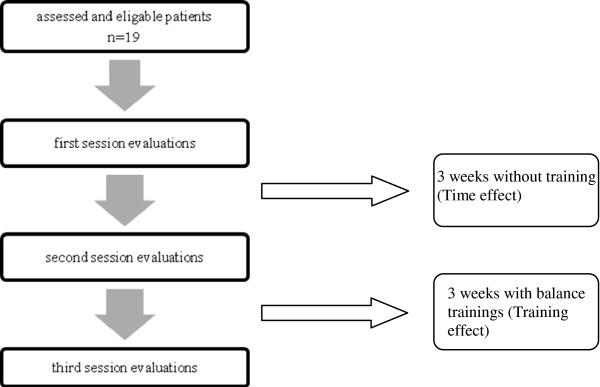
Flow chart for the method of recruiting and assessing patients.

In this study, there was a evaluation and 3-week baseline interval followed with an evaluation (time effect) and by training over 3 weeks with re-evaluation (training effect) (Figure 1). The first evaluation included descriptive information such as age, sex, height, weight, Body mass index (BMI), Valk score, diabetic history and postural strategy assessments with force platform. After the first 3-week period, patients were evaluated for the second time with Valk score and postural strategy assessments. The third evaluation after the patients had been trained by biodex balance exercises for 10 sessions in the second 3-week period again included Valk score and postural strategy assessments. At the beginning of each session, patients’ blood sugar level was tested by a glucometer to be over 7.8 mmol/lit in order to control hyper or hypoglycemia.

The Valk polyneuropathy score assessed the severity of neuropathy based on the frequency of symptom occurrence. This instrument is a 10-item sensory polyneuropathy score that has two different sub-dimensions to evaluate the sensory alteration and neuropathic pain that comprise part of the diabetic symptoms checklist-type 2 (DSC-Type 2) [26].

Force platform measurements (Kistler 9286BA; Winterthur, Switzerland) were used to evaluate the COP fluctuations. The COP is the single point location of the ground reaction force vector [27]. Reliability of COP measures has been confirmed in several previous articles [28]. Assessments were performed in upright stance by instructing the patients to stand on a force platform, touch their heels to each other with 30 degree angle between the medial borders, and focus their eyes on a marker approximately 1.5 m straight ahead while hanging their arms at their sides in comfortable position. Trials were composed of three open- eyes and three closed- eyes conditions. Each trial lasted 30 seconds with sampling rate at 50 HZ with 2 minutes rest period between each trial. The equations were coded and run, using Matlab R2007b software (Math Works Inc. Natick, MA). In the present study, total frequency power (POWER), 99% power frequency (99% FREQ), centroidal frequency (CFREQ) and frequency spectrum are reported. Total power is the integrated area of the power spectrum of COP time series and represents the mean square value of the time series. 99% power frequency is the frequency below which 99% of total power is found and shows the maximum frequency of the main power component and centroidal frequency is the frequency at which the spectral mass in concentrated [29]. Frequency spectrum was calculated by Fast Fourier Transformation (FFT) in the intervals between 0.01-0.1 Hz (low), 0.1-0.5 Hz (low-medium), 0.5-1 Hz (high-medium) and 1-3 Hz (high) of COP signals [20-24,29]. All of measures were reported for anterior-posterior (AP) and medial- lateral (ML) directions separately.

#### Training and intervention

Standing balance trainings were carried out by Biodex Stability System (Biodex 945-302; Biodex Medical Systems Inc, 20 Ramsay Rd, Shirley, New York, USA) according to balance training program in our previous study [16]. This device measures the degree of tilt about each axis of ML and AP during dynamic conditions by two under-platform potentiometers which record tilting. It moves in a 360° range of motion by a balance platform which provides up to 20° of surface tilt. At first, patients were familiarized with the device by standing on the platform with their hands at their sides. Then, in that position, the stability platform was unlocked to allow motion. Afterwards, the patients were instructed to adjust feet positions until they found a position at which they were able to maintain moving point in the center or near the center of the circles with the difficulty level of 8 (level of 8 is the easiest level and the most difficult level is 1). Then, the platform locked and the place and angles of feet were fixed for all trainings sessions. Trainings were three times a week with one day interval between each session for 30 minutes and progressed from easy to difficult by lowering the stability level through sessions with the same standing method and feet placing. Each patient started training from level 8 and progressed to more difficult and less stable levels through 10 sessions. Level progressions selected according to the quality of exercise done by patients and limits that were assigned.

Our balance training methods borrowed from reactive movement strategies and sensory strategies to provoke somatosensory information regarding the guiding effects of external visual biofeedback. For this purpose, the physical therapy team asked the patients with DN to control perturbations due to the instability and gravity effects of an unstable platform based on targets providing external visual biofeedback for balance training. These targets cued slow and small movements to provoke ankle strategy, and they indicated fast and large movements to activate hip strategy.

The training protocol consisted of three stages:

a) Limits of stability trainings were prescribed in two patterns of practices; simple and intermediate difficulty pattern. In these practices the patients were instructed to focus on visual external feedback while guiding moving point into the middle of the target. These practices required coordinated contractions of ankle muscles to make precise and small movements. Gravity accompanied with an unstable platform produce perturbations against ankle coordinated movements for pursuing and achieving the targets.

b) Weight shifting trainings were performed in three approaches. In the first approach, patients were instructed to guide moving point from quarter one to quarter three (a), In the second approach they guided moving point from quarter two to quarter four (b), and in the third approach they guided moving point in parallel to the vertical line in the feedback screen (anterior-posterior direction) (c). These practices required both ankle and hip muscles in both AP and ML directions against the perturbing effect of gravity and unstable platform. These factors affect the coordinated participation of muscles that control postural strategies and guidance of movements according to the biofeedback.

c) Stable standing practice on the BSS platform was carried out with no visual feedback and the patients instructed to focus on covered screen and hold the platform in horizontal position. Gravity in accompany with unstable platform produce perturbations against ankle coordinated muscles contractions were the result of patients estimation about their postural orientation and equilibrium.

### Data analysis

The patients’ normal distribution baseline means were analyzed with the Kolmogorov–Smirnov test. Repeated measure ANOVA was performed to test mean differences in three sessions of assessments. Mean differences of each pair of sessions were compared with the least significant differences (LSD) test. SPSS software version 15 was used for all statistical analyses.

### Sample size calculation

Based on the study by Kim et al. POWER for a healthy young women group in anterior-posterior direction of COP signals was 11.1 ± 5.3 [30] compared with 8.7 ± 4.3 in DN patients in our study. With respect to DN patients’ differences in their AP-POWER from 8.7 to 7.4 after balance trainings, the effect size was 0.6. Therefore, assuming the effect size of 0.5 (medium) to have a larger group of study, a significant alpha 0.05 and a statistical nominal power of 0.88, 19 participants were needed to test these relationships as indicated in Cohen’s Table [31].

## Results

Kolmogrov-Smirnov analysis of normality showed that all three sessions of assessments data distribution were normal.

Means and standard deviations of the scores of three sessions (Table 2) showed reduction in the Valk scores of the third session. Repeated measure ANOVA analysis indicated that within-subject effects failed to reach significance (p = 0.065).

**Table 2:** Means and standard deviations of Valk score and force platform variables in three sessions

**Parameters**	**First session**	**Second session**	**Third session**	**F value**	**P value**
Valk score	15.05 ± 7.20	15.05 ± 7.2	13.47 ± 6.09	3.865	0.065
CFREQ XO	0.08 ± 0.04	0.09 ± 0.04	0.10 ± 0.06	1.166	0.323
CFREQ XC	0.11 ± 0.06	0.12 ± 0.09	0.14 ± 0.10	0.906	0.387
CFREQ YO	0.10 ± 0.04	0.08 ± 0.03	0.08 ± 0.04	1.655	0.205
CFREQ YC	0.11 ± 0.05	0.11 ± 0.04	0.14 ± 0.09	2.049	0.144
POWER XO	8.73 ± 4.37	7.88 ± 2.94	7.48 ± 3.25	1.331	0.277
POWER XC	13.04 ± 7.58	11.42 ± 4.77	11.14 ± 6.52	1.981	0.153
POWER YO	11.49 ± 6.57	10.68 ± 5.09	8.29 ± 3.95	6.469	0.004^*^
POWER YC	13.49 ± 7.32	13.67 ± 0.66	11.70 ± 0.30	2.007	0.149
99%FREQ XO	1.65 ± 0.32	1.75 ± 0.49	1.85 ± 0.60	1.011	0.374
99%FREQ XC	1.84 ± 0.41	1.99 ± 0.52	1.90 ± 0.28	1.378	0.265
99%FREQ YO	1.52 ± 0.38	1.62 ± 0.35	1.74 ± 0.29	4.363	0.020^*^
99%FREQ YC	1.79 ± 0.40	1.86 ± 0.49	1.86 ± 0.29	0.321	0.727
P1 XO	4.33 ± 2.14	3.84 ± 1.73	3.25 ± 1.60	2.203	0.125
P1 XC	5.34 ± 4.81	4.49 ± 2.80	3.82 ± 2.97	1.719	0.194
P1 YO	5.01 ± 4.08	5.40 ± 3.03	3.46 ± 2.12	3.456	0.052
P1 YC	5.15 ± 3.69	4.90 ± 2.92	3.84 ± 2.93	2.371	0.108
P2 XO	4.03 ± 1.84	3.62 ± 1.16	3.70 ± 1.51	0.644	0.531
P2 XC	6.12 ± 2.84	5.44 ± 2.07	5.93 ± 3.64	0.873	0.426
P2 YO	6.16 ± 3.39	5.16 ± 2.63	4.14 ± 2.01	6.738	0.003^*^
P2 YC	7.68 ± 4.19	7.74 ± 4.69	6.17 ± 3.24	1.997	0.150
P3 XO	1.15 ± 1.34	0.93 ± 0.71	1.02 ± 0.85	0.687	0.509
P3 XC	2.18 ± 1.66	1.82 ± 1.40	1.85 ± 1.03	1.660	0.204
P3 YO	1.21 ± 1.03	1.02 ± 0.55	1.12 ± 0.58	0.988	0.382
P3 YC	1.58 ± 0.94	1.66 ± 1.07	1.83 ± 1.30	0.688	0.509
P4 XO	0.27 ± 0.19	0.30 ± 0.21	0.29 ± 0.25	0.549	0.583
P4 XC	0.77 ± 1.11	0.66 ± 0.48	0.64 ± 0.70	0.573	0.569
P4 YO	0.33 ± 0.22	0.38 ± 0.23	0.44 ± 0.30	3.183	0.076
P4 YC	0.85 ± 0.60	0.87 ± 0.63	0.81 ± 0.62	0.297	0.745

*XO* Anterior-posterior direction with open eyes, *XC* Anterior-posterior direction with closed eyes, *YO* Medial- lateral direction with open eyes, *YC* Medial- lateral direction with closed eyes, *POWER* Total frequency power (HZ), *99*% *FREQ* 99% power frequency (HZ), *CFREQ* centroidal frequency (HZ), P1: 0.01-0.1 (HZ), P2: 0.1-0.5 (HZ), P3: 0.5-1 (HZ), P4: 1-3 (HZ) power density. Statistically significant differences between sessions (P <0.05): asterisk.

**Figure 2:**
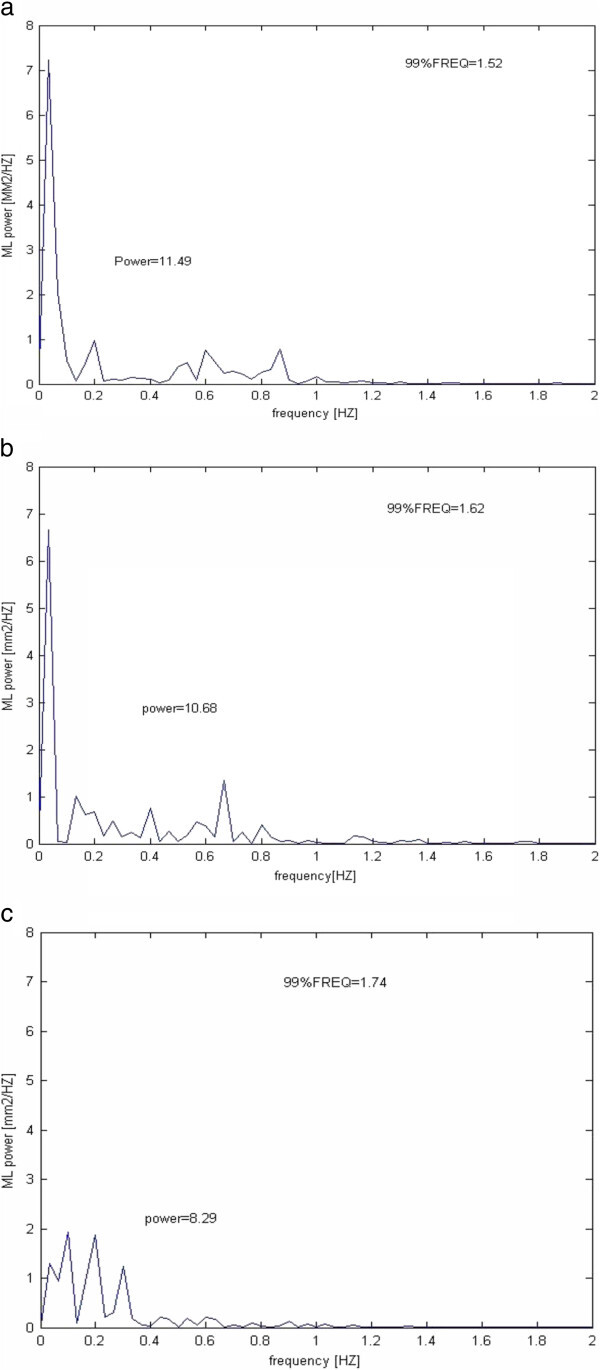
**Representative center of pressure (COP) trajectories of frequency spectrum in ML direction.** In the power spectrums, POWER and 99% FREQ are shown. **a)** first session, **b)** second session and **c)** third session.

In the analysis of Force platform variables (Table 2, Figure 2) by repeated measure ANOVA analysis, within-subject effects of open eyes conditions reached significant differences for the eyes open ML-POWER, ML-99%FREQ and ML-FFT sway power within low-medium (0.1-0.5 HZ) frequencies of COP signals while failed to reach any significant differences for other FFT sway power ranges and frequency domain analyses in AP direction or in eyes closed conditions (Table 2). The LSD test indicated significant differences for the eyes open ML- POWER and ML-FFT sway power within low-medium (0.1-0.5 HZ) frequencies of COP signals between paired sessions second and third (p = 0.019, p = 0.005), but failed to reach any significant differences between paired sessions first and second (p = 0.626, p = 0.256). Eyes open ML-99%FREQ did not show any significant differences between paired sessions in LSD test (p> 0.05).

## Discussion

In the present study, we tried to have a detailed frequency analyses about the effectiveness of balance training which should treat context-specific instabilities of DN patients’ postural control by placing more emphasis on somatosensory information. By means of these trainings, our methods recruited reactive movement strategies and sensory strategies for provoking somatosensory information with respect to the guiding contributions of external visual biofeedback. In this study, DN patients improved their balance control by reduction in eyes open ML-POWER and ML-FFT sway power within low-medium (0.1-0.5 HZ) frequencies. Furthermore, the Valk severity score did not show any significant difference in the severity of diabetic neuropathy, indicating that the better balance performance in DN patients was probably improved by other mechanisms than changes in the severity of neuropathy (Table 2).

In the present study, spectral characteristics of postural sway in DN patients were added to confirm better balance performance of DN patients. Frequency domain analyses help us to discriminate between patterns with similar time domain but different oscillation patterns, specifically, in Fourier transform [19]. In this way, the data can be expressed as a sum of simple sinusoids, each having specific frequency [19]. Some Different independent studies in Fourier transform analyses revealed that low frequencies (0.01-0.1 Hz) show visual control, the low-medium frequency band (0.1-0.5 Hz) is sensitive to vestibular stress and disturbance, the medium-high frequencies (0.5-1 Hz) link to somatosensory activity and postural reflexes mediated by lower extremities and over 1 Hz frequency band are induced by dysfunction in the central nervous system [18,20-24]. In our results we found significant reduction in eyes open ML-FFT sway power within low-medium (0.1-0.5 HZ) frequencies which is a band related to modifications in vestibular. In fact, low-medium Frequency sway is invoked in patients with peripheral vestibular pathology [21,24,32]. Moreover, Horak et al. demonstrated that DN patients with somatosensory loss, showed more dependence on vestibular information and increased vestibulospinal sensitivity as a result of somatosensory loss. Also, they implied that DN patients are unstable because the importance accredited to the vestibular system does not supply enough information for controlling balance instead of deficient somatosensory system [12]. In summary, Bonnet et al. indicated that an intact vestibular system in DN patients compensates incompletely for impairment of perceptual subsystems grounded in the somatosensory system and the efficiency of the information for about head control does not match the information from mechanoreceptors, tendons, skin and etc [5]. Thus, decrease in postural sway at eyes open low-medium frequencies (0.1-0.5 HZ) in our study shows lower reliance on vestibular system in DN patients after balance training that may show better postural control. So, DN patients get sufficient information from other subsystems such as visual or somatosensory. On the other side, the study by Oppenheim et al. who expressed precisely Fourier transform in DN patients in different bands, power of sway at medium-high frequencies (0.5-1 Hz) was reduced which is under the effects of somatosensory feedback [18]. As a result, while we did not found any differences in 0.5-1 Hz band power which may reveal that balance training had no significant improvement on somatosensory feedback, but it is probable that changes in the medium-high band power in our results provide enough information to reduce the role of vestibular subsystem and helped the CNS to appropriately integrate available information for postural control.

Lateral instability was introduced by some studies as a marker of impaired balance in DN patients [33]. Also, alterations in the balance parameters in ML direction recorded by force platform demonstrated that DN patients are less stable in the ML direction of postural sway parameters [11] and suggested the crucial role of vestibular system in the control of hip strategy in ML direction [12]. In fact, the biomechanical and sensory problems of these patients at the ankle level lead to compensation with the more available postural control information at the hip level (abductor/adductor muscles) which increases DN patients’ sensitivity to information in the ML axis [5]. Thus the reliance on the hip joint in DN patients does not result in better postural control [34]. In the present study, results exhibited decrease in eyes open ML-POWER and ML-FFT sway power within low-medium (0.1-0.5 HZ) frequencies of COP signals in DN patients after balance training (Table 2, Figure 2). Subsequently, the first possibility may be reduction in the reliance on the vestibular system which crucially controls hip strategy in ML direction. So, the modifications in the ML results shows better controlling of hip muscles after balance training. The later possibility may be related to the speculation which analyzes the higher minimum sway in DN patients as an exploratory sway and useful noise which try to pick up more useful information for postural control at the hip level. In this speculation, although increase in minimum sway negatively lead to more falls but reliance on a postural mechanism at the hip level make them to sway more in ML direction [5]. As a result, when that need provided by information from trained postural control subsystems in DN patients, the sway was decreased which was observed in our results. The study by Pripatla et al. confirmed the possibility of compensation by supplying enough information from subsensory noise in their study [35].

The last possibility was confirmed by our previous study which showed that balance training allowed patients to regain control of a degree of freedom of their hip joint [16]. Subsequently, lower usage of vestibular system and better controlling hip muscles after balance training relieve DN patients’ requirement to more exploratory sway as a compensatory strategy and showed better balance performance after balance training in DN patients. These findings are in agreement with the study by Nagy et al. which reported the effectiveness of balance training by evaluating spectral characteristics in elderly participants. The subjects showed improvement in balance confidence and in control of ML balance in response to the 8 weeks training, and the higher ML direction frequency power exhibited after the training may be indicative of better balance performance [36]. Moreover, other studies which recruited DN patients in physiotherapeutic gait and balance group training [13], structured balance exercises [14,15] confirmed the effects of balance training in DN patients.

## Conclusion

Finally, frequency and spectral analyses of postural sway in DN patients, specifically in ML direction are useful methods for evaluating postural control subsystems after balance trainings. But, further studies needs to investigate the underlying physiology of these possible alterations to precisely discriminate and decide about the role of each subsystem in the improvements after balance training.

## Abbreviations

CNS: Central nervous system; DN: Diabetic neuropathy; FBS: Fasting Blood Sugar; NCV: Nerve conduction velocity; BSS: Biodex stability system; FFT: Fast Fourier Transformation; POWER: Total frequency power; 99% FREQ: 99% power frequency; CFREQ: Centroidal frequency; AP: Anterior-posterior; ML: Medial- lateral.

## Authors' contributions

Study concept and design: HS, FB. Acquisition of data: HS, FB. Analysis and interpretation of data: HG, AE, HS. Drafting of manuscript: HS. Critical revision of manuscript for important intellectual content: HS, FB. Statistical analysis: MK. Obtained funding: FB. Administrative, technical, or material support: FB. Study supervision: HS, FB, AE. All authors read and approved the final manuscript.

## Competing of interests

The authors declare that they have no competing interests.
